# Social networks in primates: smart and tolerant species have more efficient networks

**DOI:** 10.1038/srep07600

**Published:** 2014-12-23

**Authors:** Cristian Pasquaretta, Marine Levé, Nicolas Claidière, Erica van de Waal, Andrew Whiten, Andrew J. J. MacIntosh, Marie Pelé, Mackenzie L. Bergstrom, Christèle Borgeaud, Sarah F. Brosnan, Margaret C. Crofoot, Linda M. Fedigan, Claudia Fichtel, Lydia M. Hopper, Mary Catherine Mareno, Odile Petit, Anna Viktoria Schnoell, Eugenia Polizzi di Sorrentino, Bernard Thierry, Barbara Tiddi, Cédric Sueur

**Affiliations:** 1 Université de Strasbourg, Institut Pluridisciplinaire Hubert Curien, Strasbourg, France; 2 Centre National de la Recherche Scientifique, Département Ecologie, Physiologie et Ethologie, Strasbourg, France; 3 Ecole Normale Supérieure, Paris, France; 4 University of St Andrews, Centre for Social Learning and Cognitive Evolution, School of Psychology & Neuroscience, St Andrews, United Kingdom; 5 Inkawu Vervet Project, Mawana Game Reserve, Swart Mfolozi, KwaZulu Natal, South Africa; 6 Kyoto University, Primate Research Institute, Center for International Collaboration and Advanced Studies in Primatology Kanrin 41-2, Inuyama, Aichi, Japan 484-8506; 7 Kyoto University Wildlife Research Center, 2-24 Tanaka-Sekiden-cho, Sakyo, Kyoto, Japan 606-8203; 8 Ethobiosciences, Research and Consultancy Agency in Animal Wellbeing and Behaviour, Strasbourg, France; 9 Department of Anthropology, University of Calgary, Canada; 10 University of Neuchâtel, Institute of Biology, Neuchâtel, Switzerland; 11 Department of Psychology & Language Research Center, Georgia State University, Atlanta, GA, 30302, USA; 12 Department of Anthropology, University of California, Davis, 1 Shields Ave., Davis, CA 95616, U.S.A.; 13 Smithsonian Tropical Research Institute, Ancon, Panama City, Panama; 14 Behavioral Ecology and Sociobiology Unit, German Primate Center, Göttingen, Germany; 15 Courant Research Centre “Evolution of Social Behaviour”, University of Göttingen, Germany; 16 Lester E. Fisher Center for the Study and Conservation of Apes, Lincoln Park Zoo, Chicago, IL, 60614, USA; 17 Michale E. Keeling Center for Comparative Medicine and Research, UT MD Anderson Cancer Center, Bastrop, TX, 78602, USA; 18 Unit of Social Ecology, CP231, Université libre de Bruxelles, Campus Plaine, Bd du triomphe, 1050 Brussels, Belgium; 19 Unit of Cognitive Primatology and Primate Center, ISTC-CNR, Rome, Italy; 20 Cognitive Ethology Laboratory, German Primate Center, Goettingen, Germany

## Abstract

Network optimality has been described in genes, proteins and human communicative networks. In the latter, optimality leads to the efficient transmission of information with a minimum number of connections. Whilst studies show that differences in centrality exist in animal networks with central individuals having higher fitness, network efficiency has never been studied in animal groups. Here we studied 78 groups of primates (24 species). We found that group size and neocortex ratio were correlated with network efficiency. Centralisation (whether several individuals are central in the group) and modularity (how a group is clustered) had opposing effects on network efficiency, showing that tolerant species have more efficient networks. Such network properties affecting individual fitness could be shaped by natural selection. Our results are in accordance with the social brain and cultural intelligence hypotheses, which suggest that the importance of network efficiency and information flow through social learning relates to cognitive abilities.

Networks are observed at every level of biological organisation, from molecular pathways to ecosystems^1^. The way genes, proteins and other entities interact is selected by evolutionary processes leading to so-called optimal networks^2^,^3^. For instance, gene networks have been selected to be dynamically robust to mutation, stochasticity, and changes in the environment^3^. Protein networks increase the adaptability of bacteria, which have colonised every ecological niche on earth^2^. Neural networks can approximate statistically optimal decisions^4^,^5^. Finally, at a larger scale, human communicative networks are also described as efficient when they enhance cooperation between individuals or result in improved communication and decision making^6^,^7^,^8^,^9^. Efficient networks are defined as those providing better (information) transmission in terms of speed and accuracy with the minimum number of connections between entities, because the latter are costly to build and maintain^10^,^11^. Given that network optimisation is so prevalent in biology, we expect animal social networks, through which information flows via social processes, to be selected for optimality as well.

Social networks have been increasingly studied in nonhuman animals^12^,^13^ and recent studies have shown that central individuals (i.e. those most inter-connected individuals) attain greater fitness than do peripheral ones: male chimpanzees with highest betweenness centrality coefficients have higher fitness benefits^14^, juvenile baboons with central mothers have higher survival^15^, central male beetles have higher reproductive success^16^ and central male dolphins live longer^17^. On the other hand, centrality can also have costs; for example, female centrality in Japanese macaques is positively linked with higher exposure to parasites^18^. In contrast to studies of human communication networks, very little is known about network properties in animal societies, or how these are related to efficiency at the group level. Following Waters and Fewell^19^ the network structure of interactions among ants should be selected to maximize colony-level function and/or efficiency rather than individual success, the crucial question here is whether or not social network properties enhance the fitness of group members. Living in social groups entails costs, such as competition for resources and disease and parasite transmission, in addition it also provides benefits such as predator avoidance and optimal decision making through information transmission. Social individuals therefore need to balance these costs and benefits in order to maximize their fitness within their social networks, which generates trade-offs between behavioural strategies^20^. Social transmission of behaviour and disease within a group can take many forms and may deeply affect an individual's fitness. In humans, the parasite-stress theory of sociality explains in-group assortative sociality and patterns of philopatry, xenophobia and ethnocentrism in relation to parasite pressure^21^. In theory, the social network properties, while increasing decision accuracy and information flow, should also increase the disease transmission rate creating a trade-off between decision-making efficiency and infection risk.

In many group-living animals, social transmission of information is vital for the diffusion of innovations and the maintenance of behavioural traditions^22^, for collective decision-making and coordinated motion^23^ and may have impacted the evolution of sociality along with other selective pressures such as predation or intra-group competition. Questions regarding the spread of information within groups have recently attracted much attention across disciplines, including economics^24^, social media analytics^25^ and communication^26^, and it has been shown that network structure can greatly affect the transmission process. For instance, the presence of ‘super-spreaders'^27^, as well as a high degree of community clustering (high modularity)^28^, and homophily, the formation of strong connections between similar individuals, all enhance the diffusion of information^27^.

Here, we studied network efficiency in primates. Phenomena such as culture^29^, cooperation^6^ and motion coordination^23^ (collective movements or hunting) are present in species belonging to this order and are likely to depend on social network efficiency. Moreover, there is a gradient in the social abilities of primates^30^, including humans^31^, which is linked to neocortex size^32^. Therefore, primates are an ideal subject to study the relationship between network efficiency and socio-cognitive capacities. In this study we test the influence of some group characteristics and network measures on social network efficiency. Because several studies showed that links exist between group size^31^, network density^33^ and neocortex ratio, we first predict that network efficiency should depend on mean neocortex ratio in primates. Secondly we expect that network efficiency should decrease with group size. Indeed, it has already been shown that when group size increases individuals cannot manage all social relationships inducing a decrease in network density^34^. Finally, how a social network is clustered or centralised may also affect how information is transmitted. As the presence of ‘super-spreaders'^27^ or community clustering^28^ enhance the diffusion of information^27^, we also expect these two variables to increase network efficiency.

## Methods

### Primate group data

**Figure 1:**
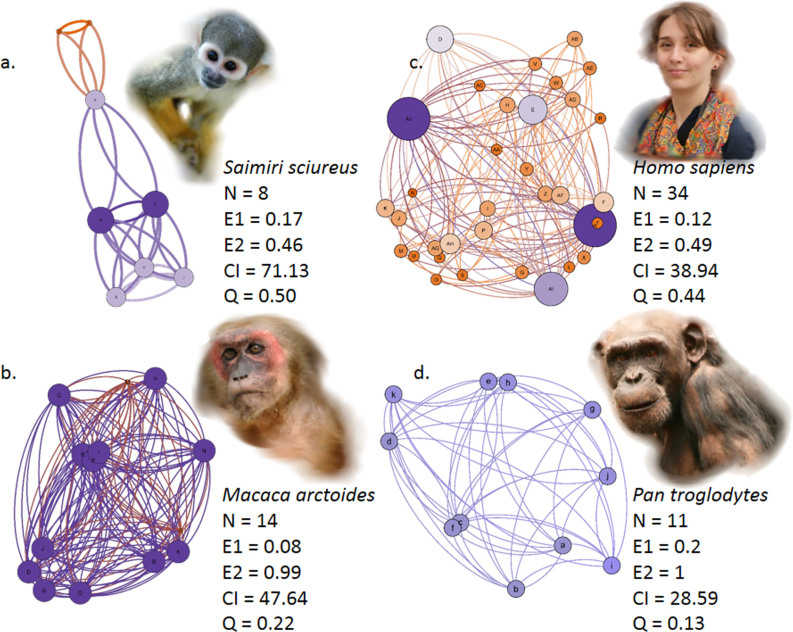
Networks of four different species depict variations between groups and network efficiencies (Global noted as E1 and Average Dyadic noted as E2). Size and colour of nodes are linked to individual centrality. The bigger and the bluer the node, the higher the centrality. CI indicates centralisation index and Q is for modularity. We chose the four groups for the variances in networks measures. As a consequence, the group size of these four examples are not representative of the mean species group size. We acknowledged C.S. for permission to use photographs.

We studied 78 groups of primates, including 4 human groups (see Supplementary Information, Extended data table 5). The dataset comprised 24 species representing 6 families and 15 genera, including 3 species of strepsirrhines (Lemurs), 5 species of platyrrhines (New World monkeys) and 16 species of catarrhines (Old World monkeys). These groups were either captive (N = 33) or free-ranging (N = 41; we considered this context as non-applicable for humans). There is no difference in group size (F_1,78_ = 3.612, P = 0.061) and in sex ratio (F_1,78_ = 0.275, P = 0.602) between captive or free-ranging groups. Network-derived data were based on socio-positive interactions (body contacts, social grooming, or proximities) and we used only one such class of interaction per group. Primate family and genus were also included in the analysis to account for variance due to phylogeny. Social networks were in all cases weighted and symmetrized in order to include the maximum number of groups for comparison (fig. 1). The neocortex ratio values used were published in previous studies^35^,^36^.

### Social network measures

We calculated two efficiency indices, plus a centralisation index, a modularity coefficient and the edge density (see Table 1), and compared them to key parameters including group size, neocortex ratio and sex ratio. Edge density is the number of observed relationships divided per the number of possible relationships; it ranges from 0 to 1 with 0 meaning that no relationships were observed and 1 that all possible relationships were observed in the group.

Efficiency is defined as how fast information can spread through the network with the minimum number of connections. It ranges from 0 to 1 with more efficient networks having values closer to 1. Following previous studies, we used two different methods to calculate network efficiency^19^,^23^,^37^,^38^: the first efficiency coefficient, named *Global Efficiency*, is the ratio between the number of individuals N, and the number of connections I multiplied by the network diameter D (the longest of the shortest paths, calculated using UCINET 6.0^39^): 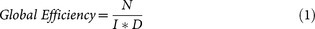
The second efficiency coefficient, named *Average Dyadic Efficiency*, is defined by computing the inverse of the shortest path length *d*, for each pair of individuals *i* and *j*, within the network (i.e. the inverse of the fewest number of links that must be used to pass from one individual to another): 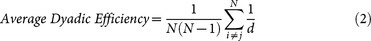
These two indices are not correlated (r = −0.17, P = 0.145) and have different meanings. While *Global Efficiency* can be interpreted as a global index (how information is transmitted from the spreader to the most peripheral individuals of the network), *Average Dyadic Efficiency* considers each pair of individuals and assesses how information should on average be transmitted between all these possible dyads (see Table 1 for detailed definitions).

In addition we calculated the *centralisation index*, which increases when the network is centralised around one or several individuals (as in the case of non-tolerant species or those which form strict hierarchies) and decreases when it is decentralised (i.e., distributed or egalitarian, as in the case of tolerant species or those with loose hierarchies)^40^,^41^. Indeed, studies have shown that individuals in tolerant species, in which the risk of injuries from conspecifics is reduced, interact with most members of the group, making individuals more connected and their network more complete. On the contrary, non-tolerant species are highly constrained by dominance and nepotism, making their network less dense and more centralised^40^,^41^,^42^. This centralisation index is derived from the eigenvector centrality *C_i_* of each individual i, the eigenvector being a measure not only of how well an individual is associated to other individuals, but also how well its neighbours are associated themselves (to have high eigenvector centrality, an individual will have relatively strong associations to other individuals with relatively strong associations): 
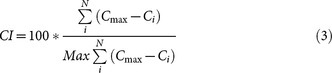
Where *CI* is the centralisation index, *C_max_* is the highest centrality and 
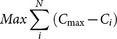
 is what the sum 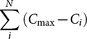 would be under the largest possible centralisation of the network (if the network was a star, centralisation index ≈ 100). The concept of centralisation is widespread in social media analytics^43^ and firm productivity related to leader management style^44^. Values higher than 100 indicate that the network is composed of isolated clusters of individuals. We assessed subgroup clustering by computing *maximum* *modularity* Q, which for any division of a group into clusters, is the fraction of internal connections in each cluster minus the expected fraction, if links were distributed at random but with the same degree sequence^28^,^45^. This maximum modularity ranged from 0 to 1, with a good estimation of group clustering (in two or several clusters) when values are close to 1. We calculated edge density, modularity and the centralisation index using respectively SocProg 2.4^46^ and UCINET 6.0^39^.

### Statistical analyses

We applied a Bayesian mixed model approach to determine which variables were significantly influencing the two efficiency response variables (see Supplementary information for statistics).

**Figure 2:**
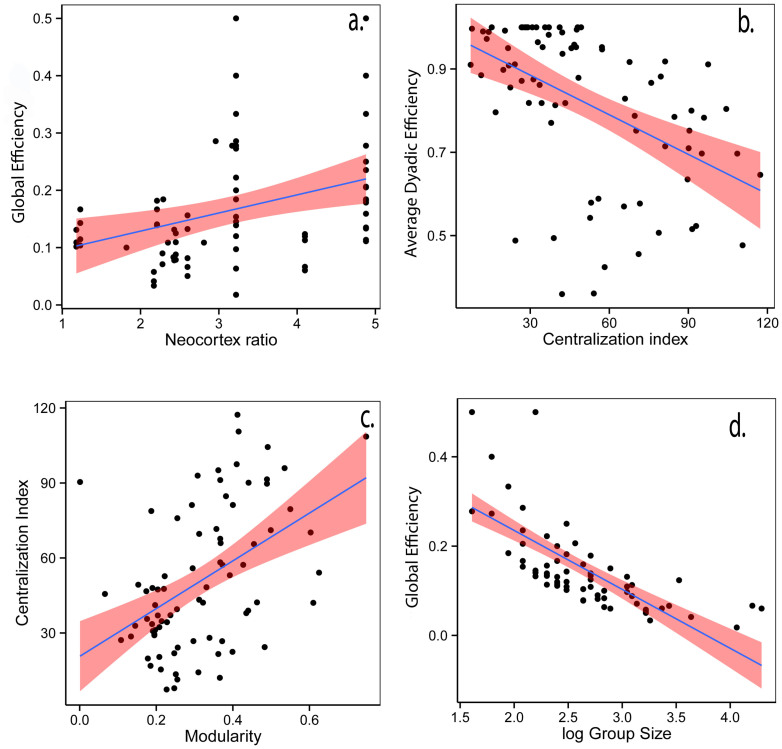
Influence of socio-species variables on network properties. All linearized models with statistical values are detailed in the supplementary information. (a.) Neocortex ratio is positively correlated with Global Efficiency. (b.) Centralisation index is negatively correlated with Average Dyadic Efficiency. (c.) Modularity is positively correlated with centralisation index. (d.) Group size is negatively correlated with Global Efficiency.

First we ran two Markov-chain Monte-Carlo (MCMC) models using the complete dataset (n = 78) to explain Global Efficiency and Average Dyadic Efficiency. Then we applied the same model procedure to a reduced dataset after removing humans from the analysis (n = 74). Since we had repeated measures for Family and Genus of the species analysed, we included them in the models as nested random effects. We used log transformed Group Size (to linearise its relationship with our efficiency measures), sex ratio (proportion of males), neocortex ratio and centralisation index as fixed effects in the models. We did not expect sex ratio to directly affect efficiency but this parameter may affect differences in social centralities between group members. Modularity and centralisation index, calculated with Ucinet 6, are both measures derived from Social Network Analysis and they are generally not independent because they reflect different aspects of the positions of individuals inside the group and are influenced by the positions of other individuals present in the network. In our dataset, modularity was positively correlated with the centralisation index (r = 0.51, P < 0.001, fig. 2c) and we therefore chose to use only the more common centralisation index for the modelling approach. We used a Pearson correlation test to examine the link between modularity and Average Dyadic Efficiency as the centralisation index influenced Average Dyadic Efficiency in the mixed models and for different other controls that cannot be tested in the main model. We applied a step-down sequential Bonferroni correction to these multiple comparisons.

## Results

### Phylogenetic variance

Before running the models we tested for homogeneity of variance in the two efficiency response variables (i.e. Global and Average Dyadic efficiencies) across the contexts of group (captive or wild) and interaction types (body contact or proximity). Variance along context and interaction type were homogeneous for both variables (Context variance test for Global Efficiency: F_1,74_ = 0.65, P = 0.200 and for Average Dyadic Efficiency: F_1,74_ = 0.95, P = 0.859; interaction type variance test for Global Efficiency: F_1,74_ = 1.63, P = 0.186 and for Average Dyadic Efficiency: F_1,74_ = 0.73, P = 0.329). Genus and family explain respectively 22.6% and 5.4% of the variance for Global Efficiency and 23.8% and less than 1% for Average Dyadic Efficiency. Phylogeny does not influence both efficiency values (Global Efficiency: K = 0.389, P = 0.673; Average Dyadic Efficiency: K = 0.318, P = 0.891) (see supplementary information for details).

### Captive versus wild context

Context – captive or wild - had an effect on density^33^ but not on efficiency. This is surprising because several studies have reported that the type of interaction and the context directly impact the strength of relationships or the range of group members^47^, and as a consequence, the structure of social networks. However, central individuals in proximity networks are also often central in contact networks, which could explain why we did not find an effect of the type of interaction on efficiency. Furthermore, captivity can reinforce social relationships because, as animals do not have to search for food, they typically spend more time engaged in social activities (with affiliative or agonistic interactions). However, an increase in social activity would influence the overall strength of the relationships, not necessarily the position of individuals within the network, which would affect efficiency. Indeed, several studies showed that individuals maintain the same ranking in centrality whatever the studied interaction (proximity, grooming or contact) even if the strength of interactions changed^48^,^49^. Other studies showed that when a group is transferred in another environment (new enclosure for instance, or from wildness to captivity), individuals are closer to each other but centralities of group members do not change; they keep the same social position in their network^50^,^51^.

### Influence of neocortex ratio

Principal relationships between variables are summarised in Extended Data Fig. 1. We found that the neocortex ratio predicted Global Efficiency; species that have a higher neocortex ratio have more efficient networks (P = 0.003, fig. 2a, Extended data table 1). This association is still present when controlling for group size (r = 0.33, P = 0.005, α = 0.006) and when excluding humans (P = 0.046, Extended data table 2). However, the neocortex ratio did not correlate with the centralisation index in our study subjects (r = 0.148, P = 0.219, α = 0.004), which means that species with higher neocortex ratio do not have more centralised networks than others.

### Effects of centralisation and modularity

Contrary to modularity (r = 0.03, P = 0.765, α_adjusted_ = 0.003), the centralisation index is well linked to group size (r = −0.23, P = 0.046, α = 0.05_adjusted_). However, when controlling for the centralisation index, Global Efficiency is still linked to group size (r = −0.76, P < 0.001, α = 0.025_adjusted_) and to neocortex ratio (r = 0.34, P = 0.002, α = 0.008_adjusted_). We suggest that either network centralisation may be explained by factors other than neocortex ratio, or that neocortex ratio affects network properties in other ways. For instance, the type of interaction does not affect the centralisation index (Context variance test: F_1,74_ = 0.80, P = 0.375) but the ecological context does (Context variance test: F_1,74_ = 4.9, P = 0.03), with wild groups having higher centralisation indices (mean = 58.31 ± 31.17) than captive ones (mean = 43.58 ± 23.18) probably due to higher food competition in the wild or to social behaviours more devoted to preferred partners (building alliances).

Contrary to our expectations, the centralisation index negatively predicted Average Dyadic Efficiency (P < 0.001, fig. 2b, Extended data table 3) but did not significantly predict Global Efficiency (P = 0.061, Extended data table 1), probably because Global Efficiency does not depend on the shortest path between each dyad but only on the diameter. Similar results were obtained with or without humans included in the dataset (see Extended data tables 2 and 4). Modularity is strongly positively correlated with the centralisation index (r = 0.51, P < 0.001, α = 0.017, fig. 2c). This correlation still exists when controlling for network density (r = 0.34, P = 0.002, α = 0.007). Here, modularity indirectly decreases efficiency (r = −0.46, P < 0.001, α = 0.0125), reinforcing findings that information and diseases have a lower probability of being transmitted between clusters in highly modular networks^52^.

### Effect of demographic variables

Sex ratios did not affect efficiencies in our models (Extended data tables 1–4). By contrast, group size affected both Global Efficiency (P < 0.001, fig. 2d and Extended data fig. 2, Extended data table 1) and Average Dyadic Efficiency (P < 0.001, Extended data table 3): the larger the group size, the lower the network efficiency. In this study, group size is not correlated with Neocortex ratio (r = −0.17, P = 0.149, α = 0.004). This is probably due to the captive groups being included in the dataset. However, even if individuals in captivity cannot “manage their group size”, they can still manage their social relationships and, as a consequence, the network efficiency. Group size is typically thought to result from a trade-off between several advantages (e.g. protection against predators and neighbouring groups, sharing of information) and disadvantages (e.g higher disease transmission, competition for resources)^53^ of sociality. Efficiency can now be added to the list of disadvantages of larger group size because it decreases the probability of information transmission across the group. Indeed, as group size increases, networks inevitably should become less dense because individuals are not able to interact with all group members^34^, and, in very large groups, they may not remember the status of their relationship with everyone in their group^33^,^35^. Indeed network density is linked to group size in our study (r = −0.41, P < 0.001, α = 0.005). Lower density networks could easily account for the apparent efficiency in these groups. Results showed that group size still affects Global Efficiency (r = −0.72, P < 0.001, α = 0.01) but not Average Dyadic Efficiency (r = 0.23, P = 0.043, α = 0.005) when controlling for density.

## Discussion

### Link between cognitive capacities and social network efficiency

The most striking result we found is the link between the neocortex ratio and efficiency. In species with higher neocortex ratio, group members should be able to adjust their social relationships in order to gain better access to social information. As an indirect consequence, this should optimize network efficiency. We know that direct structure optimisation exists in humans, for instance in transport or communication networks^7^,^8^,^11^, but this has never been demonstrated in non-human primates. We also know that brain structure reflects the complexity of social relationships. In humans, the density of grey matter and the size of the amygdala are linked to the size of social networks^31^,^54^. In non-human primates, Dunbar and colleagues have shown a link between the size of the neocortex and certain network properties (group size, density and connectivity) that relate to the number of relationships a group member is able to manage^33^,^35^. Most primate species have complex and dynamic groups resulting in daily social challenges and cognitive problems. This leads group members to develop social strategies. The more complex the network of interactions and its dynamic (involving kinship, dominance, shot-term coalitions, etc.), the more individuals have to develop complex and efficient strategies to manage this social environment. Our results provide distinctive further support for the “social brain hypothesis”^30^ because they directly link a species' relative neocortex size to the efficiency of its derived social networks. There might also be another hypothesis for which primates are not necessarily cognisant of their social network: neocortex ratio and network efficiency could be indirectly linked through social learning^29^,^55^. Species with frequent opportunities for information transmission and social learning should more readily respond to selection for moderating social relationships: if individuals can identify knowledgeable^56^ others or individuals showing fitness increasing traditions and develop relationships with these specific individuals, this could lead to an increase in network efficiency. Network ratio and network efficiency could also be linked indirectly through other factors such as ecological variables. Indeed, we might expect that some environments result in higher cognitive abilities (innovation, extractive foraging, tool use) and in efficient networks due to resources distribution shaping social relationships.

### Influence of social system on efficiency

We also found that network centralisation affects efficiency. From an evolutionary perspective, our results suggest that a more egalitarian organization, with small differences in centrality between group members, can be more efficient than a more hierarchical one^57^, suggesting a negative selection pressure on individual aggressiveness or a positive selection for tolerance of other individuals^41^. Hierarchical species might also require more redundancy in their networks (to preserve hierarchical information), increasing links between individuals and then decreasing efficiency^58^. Regarding the transmission of information, a central individual might be considered as a super-spreader, centralising information, speeding up information diffusion and decision-making. However, even if information is centralised in the apparent scale-free networks we often find approximated by animal social groups, with only one or two central individuals, the greater the number of connections, the more decentralised the network is and the faster information can be transmitted to all individuals. Studies bring contrasting results about the higher speed of information transmission in centralised versus decentralised networks^40^,^57^, but we know that decentralised networks have decreased error rates and are less prone to the diffusion of inaccurate information. Results on modularity confirm this assumption: strong differences in centrality between individuals result in more modular networks and it has been shown in humans that modularity enhances cooperation in natural^6^ and artificial^28^ groups. Modular networks, such as those found in fission-fusion societies, can therefore favour cooperation between individuals (at the cluster level) and decrease information and disease spread^59^ (at the group level).

### Evolution of social networks

**Figure 3:**
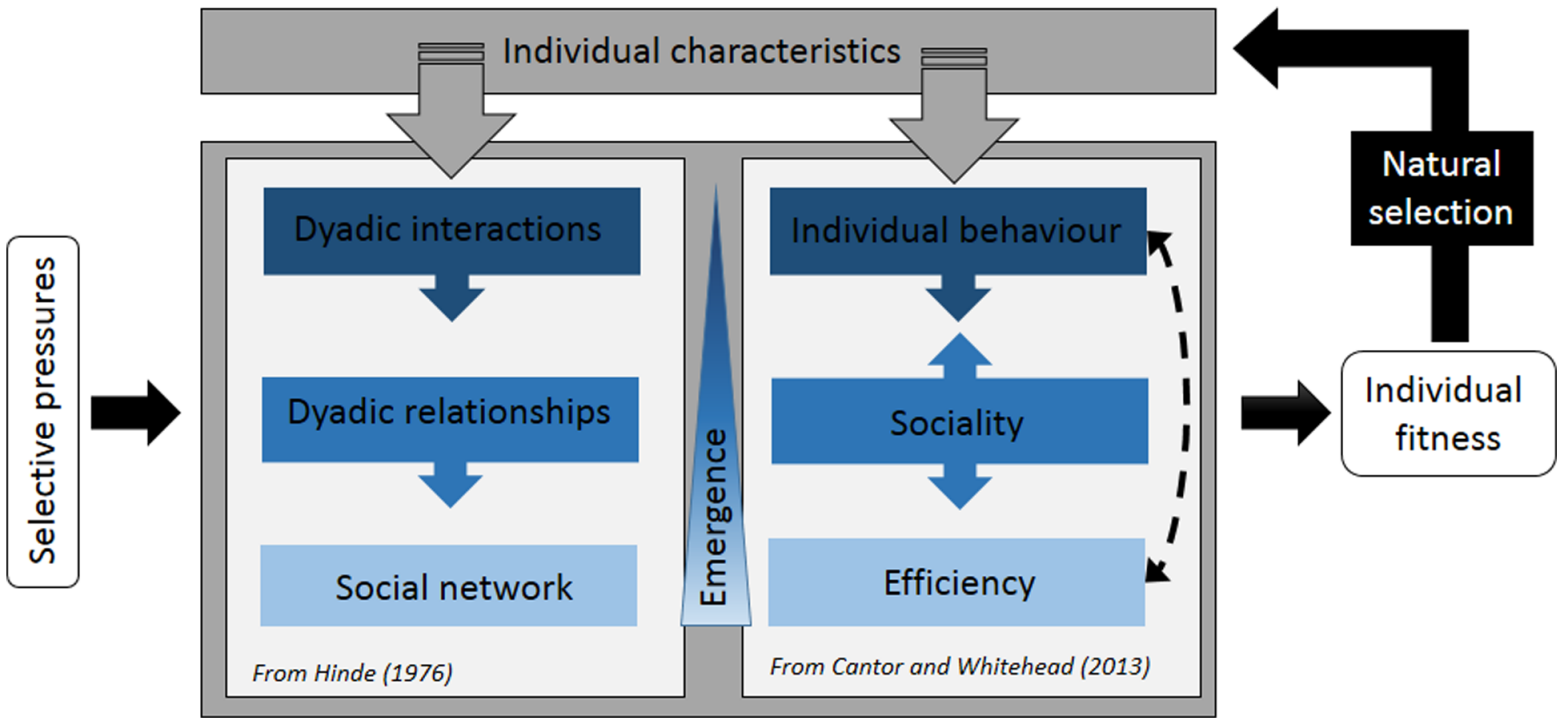
Representation of the dynamic relationship between social networks, efficiency (information or disease flow) and individuals^48^. Individual characteristics influence social networks through their effects on social relationships, and also network efficiency through variation both in individual behaviour and individual preferences for sociality. These are emergent properties because the network is more than the sum of individual interactions; therefore its properties are not directly traceable by studying only behavioural interactions. As feedback, network efficiency could influence the behaviour of individuals to be more central in the network or favour information flow. Selective pressures (ecological or social) have direct effects on how individuals interact, associate and on the overall social network, and thus on sociality and efficiency. These three different levels have a direct effect on individual fitness, which influences individual characteristics through natural selection. This overall schema shows how natural selection at the individual level can favour upper-level structure such as social networks and their efficiency.

Group members might adjust their relationships in ways that increase their fitness. In order to gain more social information, there is a pressure to be linked to a maximum number of individuals, resulting in efficient networks. On the other side, they have to be linked to related, dominant or central individuals to gain access to food or protection, resulting in more centralised or modular networks. Indeed several studies have shown that the fitness of individuals increase with their centrality^15^,^16^,^17^, suggesting the importance of being in centralized networks for these individuals. In our study, the centralisation index negatively affects efficiency (Extended data table 3), which may reflect a trade-off between increasing the network efficiency and increasing one's own centrality. On one side, networks should be decentralised to increase efficiency and information transmission; on the other side, individuals may tend to increase their own centrality (and as a consequence, the network centralisation) to gain food access, protection, grooming, etc. Another trade-off may derive from the balance between information and disease transmission^18^. Efficient social networks favour the transmission of information but also of diseases, and individuals who gain advantages by being central in information networks could also pay a cost by being central in disease networks. In the same way, but at a more global scale, efficient networks increasing information transmission should also increase transmission rate of pathogens. In a heterogeneous and unpredictable environment, group members need to share information about food resources and the network should be efficient in order to favour this information sharing. Conversely, in an environment where there is a high risk of contracting pathogens, networks should not be efficient to avoid contagion among individuals. These two trade-offs could explain the diversity of social networks observed in nature and why they are not all maximally efficient. In accordance with environmental conditions, and especially with the risks of contracting diseases or the benefits of shared information, two evolutionary forces of sociality, networks could be fashioned through individuals' behaviours and relationships in order to be more or less centralised and more or less efficient (fig. 3). Modular networks, such as those generated by fission-fusion dynamics (adaptive responses to features of the ecological and social environment), could also reflect strategies for balancing the conflict between maximizing information flow and minimizing infection risk^59^. The use of network efficiency measures to understand disease transmission could have important implications in disease management and the conservation of endangered species.

Another significant trade-off in network structure is likely to derive from achieving efficiency (due to the cost of maintaining unnecessary ties) and maintaining a suitable level of network redundancy, which is an element of robustness^58^. Networks should be expected to be robust to the loss of pathways. In animal societies like non-human primates, robustness comes in the form of maintenance of network stability despite loss of individuals or social relationships within the group. Redundancy may be particularly important for dominance interactions, because not all individuals in the network may interact (particularly in species that live in large social groups, which also tend to be the species with high social complexity and large neocortex size). Redundancy in networks may be as important as efficiency, yet redundancy and efficiency work in opposite directions: the former favours more connections, the latter favours fewer connections.

## Conclusion

Sociality is suggested to have evolved as a strategy for animals to cope with challenges in their environment. It has already been shown that social network tendencies are heritable in a gregarious primate, the rhesus macaques^42^. However, previous studies focused only on centrality at the individual-level and on the effect(s) of centrality on individuals' fitness, and not on the entire social structure and its efficiency^19^. Here, we show that network efficiency, which ultimately affects the fitness of individuals, is also linked to the neocortex ratio and to other group variables (group size, centralisation and modularity). Our results are in accordance with the cultural intelligence hypothesis^55^,^60^, which stresses the importance of the high costs of brain tissue, general behavioural flexibility and the role of social learning in acquiring cognitive skills. Our study highlights the interplay between social networks and information flow through social learning and the development of neocortex ratio (fig. 3). Network efficiency could also be selected via these mechanisms and thereby increase all group members' fitness. Species with frequent opportunities for information transmission and social learning should more readily respond to selection for managing social relationships. As for cultural complexity, species with more efficient networks should show higher cognitive abilities^55^,^60^. Future work that manipulates social network efficiency (by modifying individual centralities, information or disease flow for instance) could assess how the fitness of group members is affected and how individuals subsequently adapt their behaviours and manage their relationships to optimise their social networks within environmental constraints.

## Author Contributions

These authors contributed equally to this work: Marine Levé and Cristian Pasquaretta. All authors (C.P., M.L., N.C., E.W., A.W., A.J.J.M., M.P., M.L.B., C.B., S.F.B., M.C.C., L.M.F., C.F., L.M.H., M.C.M., O.P., A.V.S., E.P.S., B.Th., B.Ti. and C.S.) were involved in collecting data. M.L., C.P. and C.S. analysed results. M.L., C.P., M.P., A.W. and C.S. prepared the figures and tables. M.L., C.P., E.W., N.C., A.W., M.P., A.J.J.M. and C.S. wrote the paper. All authors (C.P., M.L., N.C., E.W., A.W., A.J.J.M., M.P., M.L.B., C.B., S.F.B., M.C.C., L.M.F., C.F., L.M.H., M.C.M., O.P., A.V.S., E.P.S., B.Th., B.Ti. and C.S.) were involved in commenting on the final draft of the paper. C.S supervised the study.

## Supplementary Material

Supplementary Information Supplementary information

## Figures and Tables

**Table 1:** Definitions of network indices

Network Index	Technical definition	Meaning	Instances
Global Efficiency (E1)	Ratio between the number of individuals N, and the number of connections I multiplied by the network diameter D (see equation 1)	How maximum individuals are connected with the minimum of connections	Values close to 1 indicate a minimum connection of nodes allowing optimal information transmission across a group
Average Dyadic Efficiency (E2)	Inverse of the shortest path length *d*, for each pair of individuals *i* and *j*, within the network (see equation 2)	How well information can be efficiently transmitted to all individuals	Values close to 1 allow optimal information transmission across a group
Centralisation index (CI)	Sum of the differences between each individual's centrality and the centrality of the most central individual, all divided by the sum of the differences of centralities under the hypothesis that the network was a star (see equation 3)	To what extent a network is dominated by a single or a few individuals	Values close to 0 indicate an equal or decentralised network whilst values close to 100 indicate a network centralised around one individual
Modularity (Q)	Fraction of internal connections in each cluster minus the expected fraction if connections were distributed at random but with the same degree sequence	To what extent a group is clustered	Values close to 0 indicate a purely random distribution of relationships whilst values close to 1 indicate strong hierarchical clustering
