# Supplementary material for: Social networks in primates: smart and tolerant species have more efficient networks
Source: Sci Rep. 2014 Dec 23;4:7600. doi: 10.1038/srep07600 (PMC4274513; doi:10.1038/srep07600)
Supplement: Supplementary Information [file srep07600-s1.doc]

**Supplementary information**

**Social networks in primates: smart and tolerant species have more efficient networks**

Cristian Pasquaretta, Marine Levé, Nicolas Claidière, Erica van de Waal, Andrew Whiten, Andrew J.J. MacIntosh, Marie Pelé, Mackenzie L. Bergstrom, Christèle Borgeaud, Sarah F. Brosnan, Margaret C. Crofoot, Linda M. Fedigan, Claudia Fichtel, Lydia M. Hopper, Mary Catherine Mareno, Odile Petit, Anna Viktoria Schnoell, Eugenia Polizzi di Sorrentino, Bernard Thierry, Barbara Tiddi & Cédric Sueur

1. **Supplementary methods**

**Primate Social networks**

We compiled interaction matrices for primate species following procedures similar to previous meta-analyses^1–4^. These data came from published studies or from unpublished data provided by the authors on groups already studied (Table 1). Only interactions involving close proximity were considered, including grooming, contact sitting or proximities less than one meter. We used only one kind of the above mentioned interactions per group. We preferred body contacts to proximities as this measure is more reliable and reflects more individual preference than proximity. Observation periods (in months) and time of observation (number of focal hours per group) are not correlated with measures of efficiency, densities, centrality or modularity (-0.265 < r < 0.049, P > 0.023, α = 0.005_adjusted_). We favoured social network measures allowing comparisons between groups despite the differences in data collection methods^5,6^. We compiled a total of 78 primate social networks. Details are given in Table 1.

**Phylogenetic analysis**

In order to test if the observed variation in the efficiency traits was determined by phylogenetic relationship among species we tested whether the phylogenetic signal (defined as the non independence of a trait among species) was significantly different from 0 using randomization procedure for each of the two efficiency values^7^. If, the ratio of the observed residual standard error on its randomize expected value among species, K is < 1, then the trait analysed are not significant influenced by the phylogenetic relationships. The influence of phylogenetic matrix was tested on a mean efficiency value for each species using the phylosignal function in the R package picante^7,8^. There was no influence of phylogeny on both the efficiency values (Global Efficiency: K = 0.389, P = 0.673; Average Dyadic Efficiency: K = 0.318, P = 0.891).

**Statistical analysis**

We applied a Bayesian mixed model approach (package MCMCglmm in R^9–12^) to evaluate which predictors might affect the efficiency of the network. We used the Markov-chain Monte-Carlo (MCMC) method for two main reasons. First it allows mixed models to deal with a highly unbalanced dataset (see species and family columns in Table 1) obtaining reliable estimates of the predictors contrary to what a frequentist approach does. Second it creates a Markov chain sequence that converge to a stationary posterior distribution allowing for the estimation of the mean and the 95% credibility intervals for each predictor while checking whether they overlap zero or not^10^. We specified simulation parameters in the MCMC as following: 100000 iterations, a thinning interval of 100 (i.e. only one iteration from every 100 in the Markov chain is used to estimate the posterior distribution of the parameters to reduce the autocorrelation between successive iterations) and a burn-in of 1000 to ensure convergence (the parameters are estimated after discarding the first 1000 models)^10^. We carried out multi-model inference and model average to ranking models according to their Deviance Information Criterion^11^ to evaluate the relative weight of evidence of each predictor on network efficiency using the *dredge* and *model.avg* functions in the R Package MuMIn ^13^

Global Efficiency was negatively related to group size and positively related to neocortex Ratio, respectively (Extended data table 1 in the Main Text). The same results were obtained after removing *Homo sapiens* from the analysis (Extended data table 2 in the Main Text). Average Dyadic Efficiency was negatively related both to group size and centralisation index (Extended data table 3 in the Main Text) and the results were similar when removing *Homo sapiens* (Extended data table 4 in the Main Text).

1. **Supplementary tables**

Extended data table 1 – Fixed effects for Global Efficiency model using the complete dataset. Posterior density means and their upper (U – 95% CI) and lower (L – 95% CI) credibility intervals are provided. Significant p-values (pMCMC) are presented in bold. Relative weight of evidence for each predictor is expressed as a percentage of influence on the response variable relative to other predictors tested.

|  | Posterior Mean | L – 95% CI | U – 95% CI | pMCMC | Relative weight % |
| --- | --- | --- | --- | --- | --- |
| intercept | 0.450 | 0.35 | 0.553 | **< 0.001** |  |
| Group Size | -0.135 | -0.168 | -0.01 | **< 0.001** | 100 |
| Neocortex ratio | 0.024 | 0.009 | 0.039 | **0.003** | 88 |
| Centralisation | -0.001 | -0.001 | 0.001 | 0.061 | 67 |
| Sex ratio | 0.035 | -0.043 | 0.118 | 0.388 | 25 |

Extended data table 2 – Fixed effects for Global Efficiency model excluding *Homo sapiens*. Posterior density means and their upper (U – 95% CI) and lower (L – 95% CI) credibility intervals are provided. Significant p-values (pMCMC) are presented in bold. Relative weight of evidence for each predictor is expressed as a percentage of influence on the response variable relative to other predictors tested.

|  | Posterior Mean | L – 95% CI | U – 95% CI | pMCMC | Relative weight % |
| --- | --- | --- | --- | --- | --- |
| intercept | 0.524 | 0.403 | 0.651 | **< 0.001** |  |
| Group Size | -0.152 | -0.189 | -0.116 | **< 0.001** | 100 |
| Neocortex ratio | 0.017 | 0.001 | 0.035 | **0.046** | 76 |
| Centralisation | -0.001 | -0.001 | 0.001 | 0.121 | 57 |
| Sex ratio | -0.012 | -0.11 | 0.081 | 0.812 | 28 |

Extended data table 3 – Fixed effects for Average Dyadic Efficiency model using the complete dataset. Posterior density means and their upper (U – 95% CI) and lower (L – 95% CI) credibility intervals are provided. Significant p-values (pMCMC) are presented in bold. Relative weight of evidence for each predictor is expressed as a percentage of influence on the response variable relative to other predictors tested.

|  | Posterior Mean | L – 95% CI | U – 95% CI | pMCMC | Relative weight % |
| --- | --- | --- | --- | --- | --- |
| intercept | 1.492 | 1.276 | 1.693 | **< 0.001** |  |
| Group Size | -0.152 | -0.209 | -0.09 | **< 0.001** | 100 |
| Neocortex ratio | -0.025 | -0.057 | 0.004 | 0.088 | 61 |
| Centralisation | -0.003 | -0.005 | -0.002 | **< 0.001** | 100 |
| Sex ratio | -0.049 | -0.205 | 0.086 | 0.492 | 26 |

Extended data table 4 – Fixed effects for Average Dyadic Efficiency model excluding *Homo sapiens*. Posterior density means and their upper (U – 95% CI) and lower (L – 95% CI) credibility intervals are provided. Significant p-values (pMCMC) are presented in bold. Relative weight of evidence for each predictor is expressed as a percentage of influence on the response variable relative to other predictors tested.

|  | Posterior Mean | L – 95% CI | U – 95% CI | pMCMC | Relative weight % |
| --- | --- | --- | --- | --- | --- |
| intercept | 1.413 | 1.206 | 1.636 | **< 0.001** |  |
| Group Size | -0.121 | -0.18 | -0.064 | **< 0.001** | 100 |
| Neocortex ratio | -0.014 | -0.051 | 0.023 | 0.378 | 36 |
| Centralisation | -0.004 | -0.005 | -0.003 | **< 0.001** | 100 |
| Sex ratio | -0.076 | -0.222 | 0.07 | 0.308 | 27 |

Extended data table 5**:** Dataset of the 78 primates groups

| *Group* | *Species* | *Family* | *Interaction* | *Context* | *Sex ratio* | *Group Size N* | *Number of connections I* | *Diameter D* | *Density* |
| --- | --- | --- | --- | --- | --- | --- | --- | --- | --- |
| 1 | *Alouatta palliata* | Aotidae | Proximities | Wild | 0.5 | 17 | 85 | 2 | 0.625 |
| 2 | *ateles geoffroyi* | Atelidae | Body contacts | Wild | 0.4286 | 15 | 46 | 3 | 0.438 |
| 3 | *Cebus capucinus* | Cebidae | Body contacts | Wild | 0 | 7 | 19 | 2 | 0.905 |
| 4 | *Cebus capucinus* | Cebidae | Body contacts | Wild | 0.3333 | 12 | 12 | 4 | 0.182 |
| 5 | *Cebus capucinus* | Cebidae | Body contacts | Wild | 0.5 | 14 | 22 | 4 | 0.242 |
| 6 | *Cebus capucinus* | Cebidae | Body contacts | Wild | 0.6923 | 13 | 21 | 3 | 0.269 |
| 7 | *Cebus capucinus* | Cebidae | Body contacts | Wild | 0 | 5 | 10 | 1 | 1 |
| 8 | *Cebus capucinus* | Cebidae | Body contacts | Wild | 0 | 6 | 15 | 1 | 1 |
| 9 | *Cebus capucinus* | Cebidae | Body contacts | Wild | 0 | 10 | 44 | 2 | 0.978 |
| 10 | *Saimiri sciureus* | Cebidae | Proximities | Captive | 0.25 | 8 | 16 | 3 | 0.571 |
| 11 | *Saimiri sciureus* | Cebidae | Proximities | Captive | 0.3 | 10 | 24 | 3 | 0.533 |
| 12 | *Saimiri sciureus* | Cebidae | Body contacts | Captive | 0.3333 | 12 | 66 | 1 | 1 |
| 13 | *Saimiri sciureus* | Cebidae | Proximities | Captive | 0.4444 | 9 | 16 | 4 | 0.444 |
| 14 | *Sapajus apella* | Cebidae | Body contacts | Wild | 0.1818 | 11 | 33 | 3 | 0.6 |
| 15 | *Sapajus apella* | Cebidae | Body contacts | Wild | 0.2667 | 15 | 21 | 4 | 0.2 |
| 16 | *Sapajus apella* | Cebidae | Body contacts | Wild | 0.2857 | 10 | 25 | 3 | 0.556 |
| 17 | *Sapajus apella* | Cebidae | Body contacts | Wild | 0.3 | 10 | 37 | 2 | 0.822 |
| 18 | *Sapajus apella* | Cebidae | Body contacts | Wild | 0.4 | 8 | 13 | 3 | 0.464 |
| 19 | *Sapajus apella* | Cebidae | Body contacts | Wild | 0.5 | 12 | 33 | 2 | 0.5 |
| 20 | *Sapajus apella* | Cebidae | Body contacts | Captive | 0.5714 | 7 | 21 | 1 | 1 |
| 21 | *Sapajus paella* | Cebidae | Body contacts | Wild | 0 | 5 | 9 | 2 | 0.9 |
| 22 | *Sapajus paella* | Cebidae | Body contacts | Wild | 0.25 | 8 | 17 | 2 | 0.607 |
| 23 | *Cercopithecus diana* | Cercopithecidae | Body contacts | Captive | 0.4286 | 7 | 19 | 2 | 0.905 |
| 24 | *cercopithecus mitis* | Cercopithecidae | Body contacts | Wild | 0 | 17 | 102 | 2 | 0.75 |
| 25 | *Chlorocebus pygerythrus* | Cercopithecidae | Body contacts | Wild | 0.3947 | 38 | 460 | 2 | 0.654 |
| 26 | *Chlorocebus pygerythrus* | Cercopithecidae | Body contacts | Wild | 0.4 | 25 | 217 | 2 | 0.723 |
| 27 | *Chlorocebus pygerythrus* | Cercopithecidae | Body contacts | Wild | 0.5385 | 26 | 259 | 3 | 0.797 |
| 28 | *Erythrocebus patas* | Cercopithecidae | Body contacts | Wild | 0.1111 | 8 | 28 | 1 | 1 |
| 29 | *Macaca arctoides* | Cercopithecidae | Body contacts | Wild | 0.381 | 21 | 40 | 4 | 0.19 |
| 30 | *Macaca arctoides* | Cercopithecidae | Body contacts | Captive | 0.1429 | 14 | 90 | 2 | 0.989 |
| 31 | *Macaca fuscata* | Cercopithecidae | Body contacts | Wild | 0.36 | 25 | 248 | 2 | 0.823 |
| 32 | *Macaca fuscata* | Cercopithecidae | Body contacts | Wild | 0.4194 | 31 | 117 | 4 | 0.252 |
| 33 | *Macaca fuscata* | Cercopithecidae | Body contacts | Wild | 0.4667 | 15 | 30 | 4 | 0.286 |
| 34 | *Macaca fuscata* | Cercopithecidae | Proximities | Captive | 0.3571 | 14 | 90 | 2 | 0.989 |
| 35 | *Macaca fuscata* | Cercopithecidae | Body contacts | Wild | 0.3333 | 21 | 64 | 3 | 0.305 |
| 36 | *Macaca fuscata* | Cercopithecidae | Body contacts | Captive | 0.3636 | 22 | 125 | 2 | 0.541 |
| 37 | *Macaca mulatta* | Cercopithecidae | Body contacts | Wild | 0.4375 | 16 | 49 | 4 | 0.408 |
| 38 | *Macaca mulatta* | Cercopithecidae | Body contacts | Captive | 0.2222 | 9 | 34 | 2 | 0.944 |
| 39 | *Macaca mulatta* | Cercopithecidae | Body contacts | Captive | 0.4 | 10 | 32 | 2 | 0.711 |
| 40 | *Macaca nigra* | Cercopithecidae | Body contacts | Captive | 0.375 | 16 | 98 | 2 | 0.817 |
| 41 | *Macaca radiata* | Cercopithecidae | Body contacts | Wild | 0.3478 | 23 | 162 | 2 | 0.64 |
| 42 | *Macaca radiata* | Cercopithecidae | Body contacts | Wild | 0.4375 | 16 | 89 | 2 | 0.742 |
| 43 | *Macaca tonkeana* | Cercopithecidae | Body contacts | Captive | 0.2857 | 15 | 28 | 4 | 0.267 |
| 44 | *Macaca tonkeana* | Cercopithecidae | Body contacts | Captive | 0.3 | 10 | 37 | 2 | 0.822 |
| 45 | *Macaca tonkeana* | Cercopithecidae | Body contacts | Captive | 0.48 | 25 | 231 | 2 | 0.77 |
| 46 | *Macaca tonkeana* | Cercopithecidae | Body contacts | Captive | 0.5625 | 18 | 150 | 2 | 0.981 |
| 47 | *Macaca tonkeana* | Cercopithecidae | Body contacts | Captive | 0.5714 | 10 | 45 | 1 | 1 |
| 48 | *Mandrillus sphinx* | Cercopithecidae | Body contacts | Captive | 0.2778 | 18 | 24 | 5 | 0.157 |
| 49 | *Papio ursinus* | Cercopithecidae | Body contacts | Wild | 0.2667 | 15 | 69 | 2 | 0.657 |
| 50 | *Homo sapiens* | Hominidae | Proximities | NA | 0 | 22 | 39 | 5 | 0.169 |
| 51 | *Homo sapiens* | Hominidae | Proximities | NA | 1 | 34 | 55 | 5 | 0.098 |
| 52 | *Homo sapiens* | Hominidae | Proximities | NA | 0.4138 | 29 | 240 | 2 | 0.591 |
| 53 | *Homo sapiens* | Hominidae | Proximities | NA | 0.5455 | 11 | 46 | 2 | 0.836 |
| 54 | *Pan paniscus* | Hominidae | Proximities | Captive | 0.6 | 5 | 9 | 2 | 0.9 |
| 55 | *Pan troglodytes* | Hominidae | Body contacts | Wild | 1 | 15 | 27 | 4 | 0.257 |
| 56 | *Pan troglodytes* | Hominidae | Body contacts | Wild | 0.3333 | 12 | 50 | 2 | 0.758 |
| 57 | *Pan troglodytes* | Hominidae | Body contacts | Captive | 1 | 9 | 31 | 2 | 0.861 |
| 58 | *Pan troglodytes* | Hominidae | Body contacts | Captive | 0.1667 | 5 | 10 | 1 | 1 |
| 59 | *Pan troglodytes* | Hominidae | Body contacts | Captive | 0.1818 | 11 | 55 | 1 | 1 |
| 60 | *Pan troglodytes* | Hominidae | Proximities | Captive | 0.2857 | 7 | 21 | 1 | 1 |
| 61 | *Pan troglodytes* | Hominidae | Proximities | Captive | 0.2857 | 7 | 21 | 1 | 1 |
| 62 | *Pan troglodytes* | Hominidae | Body contacts | Captive | 0.3333 | 9 | 18 | 1 | 1 |
| 63 | *Pan troglodytes* | Hominidae | Proximities | Captive | 0.3333 | 6 | 11 | 2 | 0.733 |
| 64 | *Pan troglodytes* | Hominidae | Proximities | Captive | 0.3333 | 6 | 15 | 1 | 1 |
| 65 | *Pan troglodytes* | Hominidae | Proximities | Captive | 0.375 | 8 | 26 | 2 | 0.929 |
| 66 | *Pan troglodytes* | Hominidae | Body contacts | Captive | 0.5 | 8 | 28 | 1 | 1 |
| 67 | *Pan troglodytes* | Hominidae | Body contacts | Captive | 0.5 | 10 | 45 | 1 | 1 |
| 68 | *Pan troglodytes* | Hominidae | Body contacts | Wild | 0.3276 | 58 | 1643 | 2 | 0.994 |
| 69 | *Pan troglodytes* | Hominidae | Proximities | Wild | 0.4286 | 21 | 108 | 2 | 0.514 |
| 70 | *Pan troglodytes* | Hominidae | Proximities | Captive | 0.6471 | 17 | 134 | 2 | 0.985 |
| 71 | *Pongo* | Hominidae | Proximities | Captive | 0.2 | 5 | 6 | 3 | 0.6 |
| 72 | *Eulemur catta* | Lemuridae | Proximities | Wild | 0.5 | 12 | 55 | 2 | 0.833 |
| 73 | *Eulemur catta* | Lemuridae | Proximities | Wild | 0.5 | 12 | 59 | 2 | 0.894 |
| 74 | *Eulemur catta* | Lemuridae | Proximities | Wild | 0.5455 | 11 | 42 | 2 | 0.764 |
| 75 | *Eulemur fulvus* | Lemuridae | Body contacts | Captive | 0.6364 | 11 | 48 | 2 | 0.873 |
| 76 | *Eulemur rufifrons* | Lemuridae | Proximities | Wild | 0.5833 | 12 | 42 | 2 | 0.636 |
| 77 | *Eulemur rufifrons* | Lemuridae | Proximities | Wild | 0.6364 | 11 | 33 | 2 | 0.6 |
| 78 | *Eulemur rufifrons* | Lemuridae | Proximities | Wild | 0.6429 | 14 | 45 | 3 | 0.495 |

| *Group* | *Neocortex ratio Cr* | *Global Efficiency* | *Average Dyadic Efficiency* | *Centralisation index* | *Modularity* | *Observation period (months)* | *focal hours per group* | *Reference* |
| --- | --- | --- | --- | --- | --- | --- | --- | --- |
| 1 | 1.82 | 0.1 | 0.813 | 39.5 | 0.254 | Unknown | Unknown | 14 |
| 2 | 2.35 | 0.1087 | 0.7143 | 81.2 | 0.4 | 4 | 161 | 15 |
| 3 | 4.88 | 0.1842 | 0.9524 | 34.7 | 0.214 | 5 | 110 | 16–18 |
| 4 | 4.88 | 0.25 | 0.4767 | 110.58 | 0.415 | 14 | 107 | 18,19 |
| 5 | 4.88 | 0.1591 | 0.5156 | 91.46 | 0.489 | 14 | 59 | 18,19 |
| 6 | 4.88 | 0.2063 | 0.5769 | 71.61 | 0.357 | 10 | 88 | 18,19 |
| 7 | 4.88 | 0.5 | 1 | 32.32 | 0.208 | 5 | 110 | 16–18 |
| 8 | 4.88 | 0.4 | 1 | 41.16 | 0.197 | 24 | 603 | 18,20 |
| 9 | 4.88 | 0.1136 | 0.9889 | 14.25 | 0.31 | 5 | 110 | 16–18 |
| 10 | 2.21 | 0.1667 | 0.4556 | 71.13 | 0.499 | 1 | 112 | 21 |
| 11 | 2.21 | 0.1389 | 0.7519 | 70.2 | 0.603 | 1 | 180 | 21 |
| 12 | 2.21 | 0.1818 | 1 | 28.05 | 0.339 | 1 | 11 | 8 |
| 13 | 2.21 | 0.1406 | 0.6968 | 108.56 | 0.748 | 1 | 144 | 21 |
| 14 | 4.88 | 0.1111 | 0.785 | 84.71 | 0.382 | 8 | 124 | 18,23,24 |
| 15 | 4.88 | 0.1786 | 0.523 | 92.94 | 0.308 | 8 | 73 | 18,23,24 |
| 16 | 4.88 | 0.1333 | 0.7519 | 90.39 | 0.001 | 15 | 542 | 18,25 |
| 17 | 4.88 | 0.1351 | 0.911 | 97.5 | 0.41 | 24 | 1466 | 18,26 |
| 18 | 4.88 | 0.2051 | 0.6458 | 117.29 | 0.412 | 15 | 721 | 18,25 |
| 19 | 4.88 | 0.1818 | 0.697 | 95.1 | 0.362 | 2 | 300 | 22 |
| 20 | 4.88 | 0.3333 | 1 | 37.09 | 0.237 | 1 | 11 | 8 |
| 21 | 4.88 | 0.2778 | 0.95 | 21.58 | 0.362 | 2 | 300 | 22 |
| 22 | 4.88 | 0.2353 | 0.804 | 104.38 | 0.491 | 8 | 83 | 18,23,24 |
| 23 | 2.29 | 0.1842 | 0.9524 | 57.21 | 0.373 | 6 | 88 | 27 |
| 24 | 2.42 | 0.0833 | 0.875 | 31.21 | 0.197 | 18 | Unknown | 28 |
| 25 | 2.17 | 0.0413 | 0.8184 | 29.48 | 0.195 | 24 | Unknown | 29,30 |
| 26 | 2.17 | 0.0576 | 0.8617 | 33.56 | 0.189 | 24 | Unknown | 29,30 |
| 27 | 2.17 | 0.0335 | 0.8979 | 19.78 | 0.178 | 24 | Unknown | 29,30 |
| 28 | 2.96 | 0.2857 | 1 | 26.72 | 0.372 | 1 | 259 | 31 |
| 29 | 2.43 | 0.1313 | 0.5885 | 55.88 | 0.295 | 3 | 300 | 32 |
| 30 | 2.43 | 0.0778 | 0.9945 | 47.64 | 0.22 | 28 | 191 | 33 |
| 31 | 2.6 | 0.0504 | 0.91 | 7.39 | 0.227 | 12 | 288 | 34 |
| 32 | 2.6 | 0.0662 | 0.579 | 53.09 | 0.392 | 16 | 1179 | 35 |
| 33 | 2.45 | 0.125 | 0.5699 | 65.57 | 0.455 | 12 | 764 | 36 |
| 34 | 2.45 | 0.0787 | 0.9877 | 42.135 | 0.322 | 12 | 273 | 37 |
| 35 | 2.45 | 0.1094 | 0.6349 | 89.7 | 0.489 | 12 | 140 | 38 |
| 36 | 2.45 | 0.088 | 0.7706 | 37.94 | 0.435 | 8 | 108 | 39 |
| 37 | 2.6 | 0.0816 | 0.7875 | 69.64 | 0.312 | 2 | 72 | 40 |
| 38 | 2.6 | 0.1324 | 0.9722 | 13.49 | 0.251 | 6 | 480 | 41 |
| 39 | 2.6 | 0.1563 | 0.8556 | 22.45 | 0.399 | 6 | 480 | 41 |
| 40 | Unknown | 0.0816 | 0.9083 | 21.88 | 0.247 | 8 | 289 | 39,41 |
| 41 | 2.28 | 0.071 | 0.8182 | 34.32 | 0.228 | 3 | Unknown | 43 |
| 42 | 2.28 | 0.0899 | 0.8714 | 26.75 | 0.297 | 5 | 470 | 42 |
| 43 | Unknown | 0.1339 | 0.5067 | 78.78 | 0.187 | 11 | 254 | 41,46 |
| 44 | Unknown | 0.1351 | 0.9111 | 24.16 | 0.256 | 5 | 10 | 41,45 |
| 45 | Unknown | 0.0541 | 0.885 | 11.38 | 0.255 | 6 | 120 | 41 |
| 46 | Unknown | 0.06 | 0.992 | 20.41 | 0.208 | 7 | 1000 | 41,44 |
| 47 | Unknown | 0.2222 | 1 | 15.39 | 0.212 | 7 | 1000 | 41,44 |
| 48 | Unknown | 0.15 | 0.4243 | 58.24 | 0.367 | 5 | 120 | 47 |
| 49 | 2.81 | 0.1087 | 0.8286 | 65.96 | 0.368 | 14 | 359 | 7 |
| 50 | 4.1 | 0.1128 | 0.488 | 24.35 | 0.483 | Unknown | Unknown | 49 |
| 51 | 4.1 | 0.1236 | 0.494 | 38.94 | 0.44 | 36 | Unknown | 51 |
| 52 | 4.1 | 0.0604 | 0.796 | 16.86 | 0.185 | Unknown | Unknown | 50 |
| 53 | 4.1 | 0.1196 | 0.918 | 81.18 | 0.293 | Unknown | Unknown | 48 |
| 54 | 3.22 | 0.2778 | 0.95 | 45.61 | 0.066 | 2 | 60 | 52 |
| 55 | 3.22 | 0.1389 | 0.5425 | 52.71 | 0.222 | 6 | 360 | 55,56 |
| 56 | 3.22 | 0.12 | 0.8788 | 48.3 | 0.331 | 7 | 1800 | 54 |
| 57 | 3.22 | 0.1452 | 0.9583 | 46.7 | 0.174 | 4 | 12 | 53 |
| 58 | 3.22 | 0.5 | 1 | 47.99 | 0.19 | 4 | 12 | 53 |
| 59 | 3.22 | 0.2 | 1 | 28.59 | 0.134 | 4 | 12 | 53 |
| 60 | 3.22 | 0.1842 | 0.9524 | 47.4 | 0.204 | 4 | 12 | 53 |
| 61 | 3.22 | 0.3333 | 1 | 30.78 | 0.191 | 4 | 12 | 53 |
| 62 | 3.22 | 0.5 | 1 | 35.62 | 0.176 | 4 | 12 | 53 |
| 63 | 3.22 | 0.2727 | 0.8667 | 75.91 | 0.255 | 4 | 12 | 53 |
| 64 | 3.22 | 0.4 | 1 | 29.08 | 0.195 | 4 | 12 | 53 |
| 65 | 3.22 | 0.1538 | 0.9643 | 32.87 | 0.145 | 4 | 12 | 53 |
| 66 | 3.22 | 0.2857 | 1 | 49.3 | 0.153 | 4 | 12 | 53 |
| 67 | 3.22 | 0.2222 | 1 | 27.16 | 0.108 | 4 | 12 | 53 |
| 68 | 3.22 | 0.0177 | 0.9967 | 7.89 | 0.247 | 3 | 155 | 57,58 |
| 69 | 3.22 | 0.0972 | 0.99 | 12.07 | 0.366 | 7 | 190 | 59 |
| 70 | 3.22 | 0.0634 | 0.982 | 37.01 | 0.204 | 12 | 466 | 6 |
| 71 | 3.17 | 0.2778 | 0.783 | 95.94 | 0.535 | 2 | 50 | 52 |
| 72 | 1.18 | 0.1091 | 0.9167 | 67.69 | 0.367 | 2 | 286 | 60 |
| 73 | 1.18 | 0.1017 | 0.947 | 57.26 | 0.427 | 3 | 171 | 60 |
| 74 | 1.18 | 0.131 | 0.8818 | 79.54 | 0.55 | 2 | 276 | 60 |
| 75 | 1.23 | 0.1146 | 0.9364 | 42.23 | 0.463 | 4 | 18 | 61 |
| 76 | 1.23 | 0.1429 | 0.8182 | 43.29 | 0.312 | 10 | 110 | 62 |
| 77 | 1.23 | 0.1667 | 0.8 | 91.17 | 0.367 | 10 | 129 | 62 |
| 78 | 1.23 | 0.1037 | 0.7097 | 90.11 | 0.441 | 10 | 120 | 62 |

1. **Supplementary Figures**


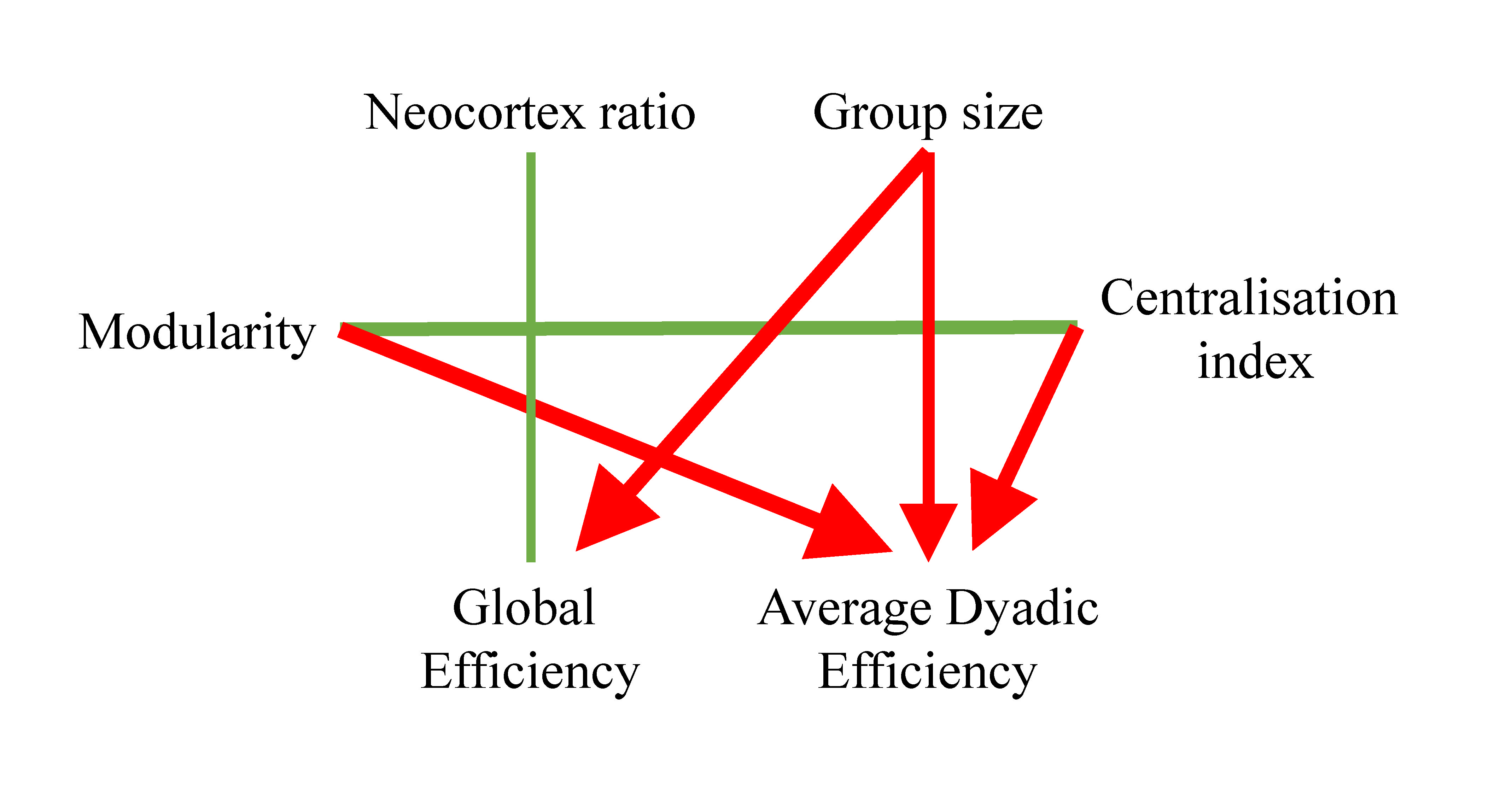


Extended data figure 1: Principal relationships between variables. Green lines indicate positive correlations between variables and red lines indicate negative correlations. An arrow indicates a causal relationship between variables. Thickness of lines vary according to strength of relationships between variables.


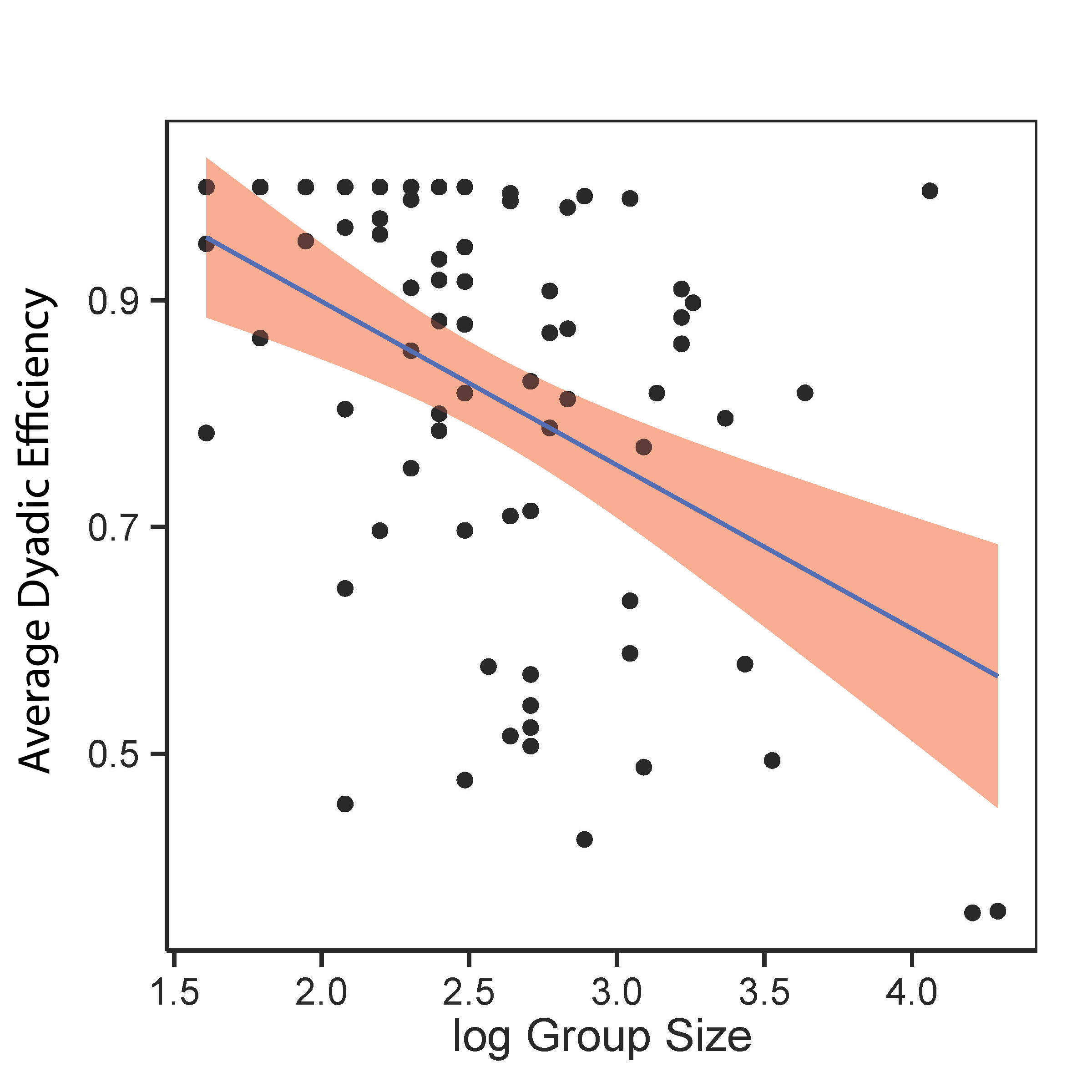


Extended data figure 2: Relationship between log(group size) and Average Dyadic Efficiency.

**References**

1. Griffin, R. H. & Nunn, C. L. Community structure and the spread of infectious disease in primate social networks. *Evol. Ecol.* **26,** 779–800 (2012).

2. Kudo, H. & Dunbar, R. I. M. Neocortex size and social network size in primates. *Anim. Behav.* **62,** 711–722 (2001).

3. Lehmann, J. & Dunbar, R. I. M. Network cohesion, group size and neocortex size in female-bonded Old World primates. *Proc. R. Soc. B Biol. Sci.* **276,** 4417 –4422 (2009).

4. Schino, G. Grooming, competition and social rank among female primates: a meta-analysis. *Anim. Behav.* **62,** 265–271 (2001).

5. Sueur, C. *et al.* A comparative network analysis of social style in macaques. *Anim. Behav.* **82,** 845–852 (2011).

6. Faust, K. & Skvoretz, J. Comparing Networks Across Space and Time, Size and Species. *Sociol. Methodol.* **32,** 267–299 (2002).

7. Blomberg, S. P., Garland, T. & Ives, A. R. Testing for Phylogenetic Signal in Comparative Data: Behavioral Traits Are More Labile. *Evolution* **57,** 717–745 (2003).

8. Kembel, S. W. *et al.* Picante: R tools for integrating phylogenies and ecology. *Bioinformatics* **26,** 1463–1464 (2010).

9. R Development Core Team. *R: A language and environment for statistical computing*. (R Foundation for Statistical Computing, 2009).

10. Hadfield, J. D. MCMC methods for multi-response generalized linear mixed models: the MCMCglmm R package. *J. Stat. Softw.* **33,** 1–22 (2010).

11. Gelman, A. *et al.* *Bayesian Data Analysis, Third Edition*. (CRC Press, 2013).

12. Smith, R. L. & Naylor, J. C. A Comparison of Maximum Likelihood and Bayesian Estimators for the Three- Parameter Weibull Distribution. *Appl. Stat.* **36,** 358 (1987).

13. Barton, K. & Barton, M. K. Package ‘MuMIn’. *Version* **1,** 18 (2013).

14. J Froehlich & Thorington, R. 1982. in *Ecol. Trop. For. Seas. Rhythms Long-Term Chang.* 291–305

15. Ahumada, J. A. Grooming behavior of spider monkeys (Ateles geoffroyi) on barro colorado Island, Panama. *Int. J. Primatol.* **13,** 33–49 (1992).

16. Bergstrom, M. L. & Fedigan, L. M. Dominance among female white-faced capuchin monkeys (Cebus capucinus): hierarchical linearity, nepotism, strength and stability. *Behaviour* **147,** 899–931 (2010).

17. Bergstrom, M. L. & Fedigan, L. M. Dominance style of female white-faced capuchins. *Am. J. Phys. Anthropol.* **150,** 591–601 (2013).

18. Polizzi, E. *et al.* Comparing Affiliative Relationships of Cebus and Sapajus Species: A Social Networking Approach. in *Folia Primatol. (Basel)* **84,** 313–313 (KARGER ALLSCHWILERSTRASSE 10, CH-4009 BASEL, SWITZERLAND, 2013).

19. Crofoot, M. C., Rubenstein, D. I., Maiya, A. S. & Berger-Wolf, T. Y. Aggression, grooming and group-level cooperation in white-faced capuchins (Cebus capucinus): insights from social networks. *Am. J. Primatol.* **73,** 821–833 (2011).

20. Perry, S. Female-female social relationships in wild white-faced capuchin monkeys,Cebus capucinus. *Am. J. Primatol.* **40,** 167–182 (1996).

21. Vaitl, E. A. Experimental analysis of the nature of social context in captive groups of squirrel monkeys (Saimiri sciureus). *Primates* **18,** 849–859 (1977).

22. Izawa, K. Social behavior of the wild black-capped capuchin (Cebus apella). *Primates* **21,** 443–467 (1980).

23. Tiddi, B., Aureli, F., Sorrentino, E. P. di, Janson, C. H. & Schino, G. Grooming for tolerance? Two mechanisms of exchange in wild tufted capuchin monkeys. *Behav. Ecol.* **22,** 663–669 (2011).

24. Tiddi, B., Aureli, F., Schino, G. & Voelkl, B. Social relationships between adult females and the alpha male in wild tufted capuchin monkeys. *Am. J. Primatol.* **73,** 812–820 (2011).

25. Izar, P. *et al.* Flexible and conservative features of social systems in tufted capuchin monkeys: comparing the socioecology of Sapajus libidinosus and Sapajus nigritus. *Am. J. Primatol.* **74,** 315–331 (2012).

26. Verderane, M. P., Izar, P., Visalberghi, E. & Fragaszy, D. M. Socioecology of wild bearded capuchin monkeys (Sapajus libidinosus): an analysis of social relationships among female primates that use tools in feeding. *BEHAVIOUR* **150,** 659–689 (2013).

27. Byrne, R. W., Conning, A. M. & Young, J. Social relationships in a captive group of Diana monkeys (Cercopithecus diana). *Primates* **24,** 360–370 (1983).

28. Rowell, T. E., Wilson, C. & Cords, M. Reciprocity and partner preference in grooming of female blue monkeys. *Int. J. Primatol.* **12,** 319–336 (1991).

29. Borgeaud, C., van de Waal, E. & Bshary, R. Third-Party Ranks Knowledge in Wild Vervet Monkeys (Chlorocebus aethiops pygerythrus). *PLoS ONE* **8,** e58562 (2013).

30. Waal, E. van de, Borgeaud, C. & Whiten, A. Potent Social Learning and Conformity Shape a Wild Primate’s Foraging Decisions. *Science* **340,** 483–485 (2013).

31. Nakagawa, N. Distribution of affiliative behaviors among adult females within a group of wild patas monkeys in a nonmating, nonbirth season. *Int. J. Primatol.* **13,** 73–96 (1992).

32. Estrada, A., Estrada, R. & Ervin, F. Establishment of a free-ranging colony of stumptail macaques (Macaca arctoides): social relations I. *Primates* **18,** 647–676 (1977).

33. Dow, M. M. & de Waal, F. Assignment methods for the analysis of network subgroup interactions. *Soc. Netw.* **11,** 237–255 (1989).

34. Jaman, M. F. & Huffman, M. A. Enclosure environment affects the activity budgets of captive Japanese macaques (Macaca fuscata). *Am. J. Primatol.* **70,** 1133–1144 (2008).

35. MacIntosh, A. J. J. *et al.* Monkeys in the Middle: Parasite Transmission through the Social Network of a Wild Primate. *PLoS ONE* **7,** e51144 (2012).

36. Furuichi, T. Inter-male associations in a wild Japanese macaque troop on Yakushima Island, Japan. *Primates* **26,** 219–237 (1985).

37. Corradino, C. Proximity structure in a captive colony of Japanese monkeys (Macaca fuscata fuscata): an application of multidimensional scaling. *Primates* **31,** 351–362 (1990).

38. Takahashi, H. & Furuichi, T. Comparative study of grooming relationships among wild Japanese macaques in Kinkazan A troop and Yakushima M troop. *Primates* **39,** 365–374 (1998).

39. Petit, O., Abegg, C. & Thierry, B. A Comparative Study of Aggression and Conciliation in Three Cercopithecine Monkeys (Macaca fuscata, Macaca nigra, Papio papio). *Behaviour* **134,** 415–432 (1997).

40. Sade, D. S. Sociometrics of Macaca Mulatta III: n-path centrality in grooming networks. *Soc. Netw.* **11,** 273–292 (1989).

41. Sueur, C. *et al.* A comparative network analysis of social style in macaques. *Anim. Behav.* **82,** 845–852 (2011).

42. Sugiyama, Y. Characteristics of the social life of bonnet macaques (Macaca radiata). *Primates* **12,** 247–266 (1971).

43. Koyama, N. Dominance, grooming, and clasped-sleeping relationships among bonnet monkeys in India. *Primates* **14,** 225–244 (1973).

44. De Marco, A. Conflicts induce affiliative interactions among bystanders in a tolerant species of macaque (Macaca tonkeana). *Anim. Behav.* **80,** 197–203 (2010).

45. Sueur, C., Petit, O. & Deneubourg, J. Selective mimetism at departure in collective movements of Macaca tonkeana: an experimental and theoretical approach. *Anim. Behav.* **78,** 1087–1095 (2009).

46. Petit, O. & Thierry, B. Aggressive and peaceful interventions in conflicts in Tonkean macaques. *Anim. Behav.* **48,** 1427–1436 (1994).

47. Petit, O., Bret, C., Sueur, C., Verrier, D. & Deneubourg, J.-L. Social Structure of a Semi-Free Ranging Group of Mandrills Mandrillus sphinx: What Role for Central Individuals? in *Folia Primatol. (Basel)* **84,** 311–311 (KARGER ALLSCHWILERSTRASSE 10, CH-4009 BASEL, SWITZERLAND, 2013).

48. B, E. Experimental investigations of childhood play. *Psychol. Bull.* **31,** 47–66 (1934).

49. Schwimmer, E. G. Exchange in the social structure of the Orokaiva. (1970).

50. Vickers, M. & Chan, S. *Representing classroom social structure*. (Melbourne: Victoria Institute of Secondary Education, 1981).

51. Zachary, W. W. An information flow model for conflict and fission in small groups. *J. Anthropol. Res.* 452–473 (1977).

52. Pelé, M., Dufour, V., Thierry, B. & Call, J. Token transfers among great apes (Gorilla gorilla, Pongo pygmaeus, Pan paniscus, and Pan troglodytes): Species differences, gestural requests, and reciprocal exchange. *J. Comp. Psychol.* **123,** 375–384 (2009).

53. Silk, J. B., Brosnan, S. F., Henrich, J., Lambeth, S. P. & Shapiro, S. Chimpanzees share food for many reasons: the role of kinship, reciprocity, social bonds and harassment on food transfers. *Anim. Behav.* **85,** 941–947 (2013).

54. Sugiyama, Y. & Koman, J. Social structure and dynamics of wild chimpanzees at Bossou, Guinea. *Primates* **20,** 323–339 (1979).

55. Sugiyama, Y. Social behavior of chimpanzees in the Budongo Forest, Uganda. *Primates* **10,** 197–225 (1969).

56. Sugiyama, Y. Social organization of chimpanzees in the Budongo Forest, Uganda. *Primates* **9,** 225–258 (1968).

57. Shimada, M. Wild chimpanzees can perform social grooming and social play behaviors simultaneously. *Primates* **54,** 315–317 (2013).

58. Shimada, M. Dynamics of the temporal structures of playing clusters and cliques among wild chimpanzees in Mahale Mountains National Park. *Primates* **54,** 245–257 (2013).

59. SCHEL, A. *et al.* Network Analysis of Social Changes in a Captive Chimpanzee Community Following the Successful Integration of Two Adult Groups. *Am. J. Primatol.* **75,** 254–266 (2013).

60. Koyama, N., Ichino, S., Nakamichi, M. & Takahata, Y. Long-term changes in dominance ranks among ring-tailed lemurs at Berenty Reserve, Madagascar. *Primates* **46,** 225–234 (2005).

61. Jacobs, A., Sueur, C., Deneubourg, J. L. & Petit, O. Social Network Influences Decision Making During Collective Movements in Brown Lemurs (Eulemur fulvus fulvus). *Int. J. Primatol.* **32,** 721–736 (2011).

62. Kappeler, P. M. & Fich℡, C. Female reproductive competition in Eulemur rufifrons: eviction and reproductive restraint in a plurally breeding Malagasy primate. *Mol. Ecol.* **21,** 685–698 (2012).
